# The roles of French community pharmacists in palliative home care

**DOI:** 10.1186/s12904-024-01406-6

**Published:** 2024-03-23

**Authors:** Isabelle Cuchet, Michael Dambrun, Sabrina Bedhomme, Chantal Savanovitch, Hélène Vaillant Roussel, Axelle Maneval

**Affiliations:** 1 Laboratory of Social and Cognitive Psychology (LAPSCO) (LAPSCO), UMR CNRS 6024, University Clermont Auvergne, Clermont-Ferrand, 63000 France; 2 UR ACCePPT, University Clermont Auvergne, Clermont-Ferrand, 63000 France; 3 CHU Clermont-Ferrand, Palliative Care Unit, Clermont-Ferrand, France

**Keywords:** Palliative home care, Community pharmacists, End-of-life support, Relational continuity, Compliance

## Abstract

**Background:**

The World Health Organization identifies pharmacists as a key resource in palliative care. However, the roles of these professionals in end-of-life care at home remain poorly understood, and community pharmacists themselves sometimes struggle to recognize their true role in this care. The aim of our study was to analyze community pharmacists’ representations of their roles in palliative care at home in France.

**Methods:**

The methodology was qualitative and based on semi-structured interviews with community pharmacists (*n* = 26). The analysis of the interviews was carried out using a qualitative content approach with thematic and lexical analysis.

**Results:**

Three main elements of the community pharmacist’s role were identified: drug expertise, care management, and psychosocial support for patients and their families.

**Conclusions:**

This study highlights a wide variety of roles adopted by French community pharmacists in palliative care at home. Some of these roles, which are in line with WHO recommendations on palliative care, have been little described to date. These roles of community pharmacists in home-based palliative care could be better recognized, and the players better integrated into end-of-life care systems at home, in order to improve such care.

**Trial registration:**

This work was carried out within the framework of a call for projects from the Fondation de France and has received the approval of the University Clermont Auvergne Research Ethics Committee (no. IRB00011540-2021-60).

**Supplementary Information:**

The online version contains supplementary material available at 10.1186/s12904-024-01406-6.

## Background

Forty million people worldwide need palliative care, but only 14% of them actually receive it [1]. Due to the increasing burden of non-communicable diseases and aging populations, the need for palliative care is expected to increase in the coming years. The World Health Organization (WHO) states that “adequate national policies, programmes, resources, and training on palliative care among health professionals are urgently needed in order to improve access” [1]. Physicians, nurses, support workers, allied health professionals, physiotherapists, and pharmacists are identified by the global organization as key resources for palliative care.

Pharmacists in particular are identified as pillars of end-of-life care. Recently, Geiger et al. [2] examined the evolution of the pharmacist’s role in palliative care in the United States since the 1980s. The authors described a professional who has evolved from a provider of therapeutics 40 years ago to an expert in medication and pain, nausea and vomiting, integrated into specialized multidisciplinary teams, with significant responsibility for adapting treatments and dosages and direct involvement in the practice of sedation. The American Society of Health-System Pharmacists (ASHP), meanwhile, has provided recommendations for pharmacists working in palliative care [3]. Its authors point to extremely varied roles and responsibilities, with both a clinical component, including direct patient care, prescription review and adjustment, and therapeutic education, and a more administrative component related to medication management. However, there is still some uncertainty about the specific role of community pharmacists, whose patients still live at home [4, 5]. In most Western countries, the home is becoming a priority place for palliative care at the request of patients who wish to “end their lives at home” [6], with public authorities eager to relieve hospital pressure. In this context, community pharmacists could become important players in end-of-life care. A recent qualitative study [7] showed that awareness of their role among community pharmacists is not easy to achieve. The main aim of the present study was to examine the community pharmacists’ representations of their role in palliative home care in France.

## Methods

### Type of study and sample

The study is qualitative research. It is inspired by the interpretative phenomenological approach (IPA) [8], a reference method in qualitative research for exploring how a life experience was felt and understood by the person who lived it [9]. Our purpose was to investigate experiences that grounded in community pharmacists with the main research question: “What are the roles of community pharmacists dealing with home palliative care?”. As low previous studies on this subject have been published yet, we wanted to explore how these professionals understood their experience in this field. The study is based on individual interviews with volunteer community pharmacists at their pharmacy. A purposive sample with maximum variation was chosen. The objective was to select community pharmacists working in a variety of pharmacy settings to reflect the diversity of professionals’ experiences with palliative care. Participants were recruited based on pre-selected characteristics. An initial hypothesis was that the working context of the community pharmacists interviewed—rural area, urban area, and commercial area—is an important factor contributing to professionals’ perceived roles in home palliative care. Targeted pharmacies were thus selected for the survey primarily on the basis of practice area (rural, urban, or mall pharmacy). Participants were recruited as the study progressed. A total of 66 community pharmacists from the Auvergne-Rhône-Alpes region were contacted first by e-mail and then by telephone between 7 April and 20 July 2021 to obtain 27 appointments. At the first contact, the investigator introduced herself as a researcher in psychology. After a short introduction describing the subject of the study, community pharmacists were asked to meet at their pharmacy. The investigator specified that the survey was pseudonymized, so that the participant and his patients could not be identified. Data saturation was considered to have been reached when the interviews no longer carried information useful for the emergence of a new role or experience among the professionals interviewed. Data saturation was established after 26 interviews.

Data were collected between 21 April and 5 August 2021. A single interview took place with each participant. When she arrived at the pharmacy, the investigator was generally taken to a confidential area of the pharmacy. If this was not the case, she asked the participant if the interview could take place in a private area. The interview took place face-to-face between the interviewer and the community pharmacist but could be interrupted by colleagues or by telephone. An interview grid that had been pretested with three community pharmacists in a pilot study was used to conduct the interviews with each participant (see Supplementary file, interview grid). Sociodemographic data collected from the interviews included age, gender, professional status (pharmacy holder/pharmacist assistant), geographic area of practice (rural pharmacy, urban pharmacy, mall pharmacy), size of the pharmacy based on number of employees, the number of years of experience of the participant, whether he or she had training in palliative care or home care, and the estimated number of times he or she had managed palliative or end-of-life care patients at home in the previous six months. The investigator took notes during the interviews which were recorded and transcribed for analysis after anonymization. These transcriptions were not sent to participants for validation.

### Analysis of the data

The transcriptions were analysed using a qualitative content approach with thematic and lexical analysis. Thematic analysis of the verbatim reports followed two phases: an exploratory reading of the interview transcripts was first carried out by the investigator; then a thematic rereading was carried out using Excel and NVivo 1.6.1 software. This software facilitates the coding of verbatim according to themes chosen by the investigator. The coding tree was discussed within the interviewer and an Associate Professor. A lexical analysis was finally launched in an automated way with Alceste software (IMAGE, CNRS, France; for an example of an analysis done with Alceste, see [10]). This software performs a statistical analysis of textual data by searching for the distribution laws of the data within a digitized corpus [11, 12]. The tool highlights the links between words (verbs or nouns) in text segments and constructs tables of term co-presence, which makes it possible to structure the corpus into lexical classes of similar context [13]. This lexical analysis is used alone or can be used as a complementary tool to thematic or other analysis (for an example, see [14]).

## Results

### Participants’ profiles

Of the 26 interviews conducted, one interview could not be used due to a technical error. The 25 interviews analyzed lasted on average 53’32’’ (*SD* = 11’22’’). The main elements of the participants’ profiles are reported in Table 1. The participants (all community pharmacists) were five pharmacists’ assistants (employees of holders) and 21 pharmacy holders, 8 men and 18 women, aged between 20 and 70 years (*M* = 46.2 years, *SD* = 11.2 years). They had been in their role for between eight months and 38 years, (*M* = 17.8 years, *SD* = 10.8 years). Ten interviews took place in rural areas, eight of which were in municipalities with fewer than 5,000 inhabitants and two in municipalities with between 5,000 and 10,000 inhabitants; 16 interviews took place in urban areas (neighborhood pharmacies and shopping mall pharmacies), six of which were in municipalities with between 10,000 and 100,000 inhabitants, and ten in municipalities with more than 100,000 inhabitants. The number of cases of end-of-life or palliative care identified by participants during the past six months ranged from zero to 35 (*M* = 5.6, *SD* = 7.71).

**Table 1:** Sociodemographic characteristics of participants

Participant	Hierarchic level	Age range	Years of experience	Type of area	Level of medical supply*	Number of pharmacy staff**	Number of cases of end-of-life or palliative care identified during the last 6 months
1	Holder	31/40	1.5	Rural area	0	2	0
2	Holder	51/60	27	Rural area	1	3	35
3	Holder	41/50	15	Urban area	2	2	7
4	Holder	31/40	1	Urban area	2	2	2
5	Holder	61/70	25	Urban area	2	2	2
6	Holder	41/50	22	Rural area	2	3	10
7	Holder	51/60	29	Rural area	0	2	3
8	Holder	41/50	18	Urban area	2	2	2
9	Holder	51/60	20	Urban area	2	1	0
10	Holder	51/60	38	Urban area	2	1	3
11	Assistant	21/30	1	Urban area	1	3	7
12	Holder	51/60	29	Urban area	2	2	2
13	Holder	51/60	15	Urban area	2	2	1
14	Assistant	51/60	23	Urban area	1	2	3
15	Holder	41/50	-	Mall area	1	3	10
16	Holder	41/50	17	Rural area	1	3	5
17	Holder	51/60	30	Urban area	2	2	0
18	Holder	51/60	24	Rural area	1	2	20
19	Holder	41/50	3	Mall area	2	3	1
20	Assistant	31/40	0.08	Mall area	1	3	2
21	Assistant	31/40	7	Urban area	1	3	2
22	Holder	41/50	25	Rural area	1	2	10
23	Holder	41/50	20	Mall area	2	3	10
24	Holder	31/40	10	Mall area	2	3	3
25	Assistant	21/30	1	Urban area	1	3	0

*Note*: *Level of medical supply: 0 = fewer than 2.5 consultations per inhabitant per year; 1 = between 2.5 and 4 consultations per inhabitant per year; 2 = more than four consultations per inhabitant per year; national average = 3.93 consultations per inhabitant per year. ** Number of pharmacy staff: 1 = less than 3 people; 2 = between 3 and 7 people; 3 = more than 7 people; national average = 4 people

### Types of end-of-life care described

At the beginning of the interview, the interviewer asked the participant to detail in a chronological sequence his or her last care of a patient in palliative care at home (see Supplementary file, interview grid). The average age of the patients was 71 years (SD = 14,77) (Table 2). In 17 situations, cancer was the identified cause of the patient’s entry into palliative care. One participant had no recollection of an experience of managing an end-of-life situation. In ten situations, management was still ongoing. In three interviews, the death had occurred less than one month ago. In ten interviews, the death had taken place between one month and one year ago. Finally, in one interview, the death had occurred more than one year ago.

**Table 2:** Palliative or end-of-life care situations and role highlights

Participant	Age range of patient	Pathology or reason given for entering end of life	Professional or structure in charge of the medical follow-up	Temporality of the care	Highlights of the role						
					Pain relief	Nutrition support	Home delivery	Psychosocial support for the entourage	Psychosocial support for the patient	Therapeutic education	Vigilance on compliance
1	71/80	Unknown	General practitioner	8 months ago	x		x	x	x	x	
2	81/90	Cancer	General practitioner	Present	x	x	x	x	x		
3	91/100	Old age	General practitioner	Present		x	x	x		x	x
4	61/70	Cancer	General practitioner	6 months ago	x	x	x	x	x	x	
5	81/90	Polypathology	Hospital	These last months	x	x	x	x	x	x	
6	71/80	Cancer	Home hospital	Present	x	x	x	x			
7	61/70	Cancer	Home hospital	Present	x	x	x		x		x
8	41/50	Cancer	Hospital	Present		x	x	x	x	x	x
9	91/100	Old age	General practitioner	Last 6 months			x		x	x	x
10	unknown	Cancer	Home hospital	Present	x	x	x		x		
11	71/80	Cancer	Hospital	1.5 months ago	x	x	x	x	x	x	x
12	41/50	Cancer	Hospital	A little more than one year	x	x	x	x	x	x	
13	41/50	Cancer	Home hospital	Present	x		x		x		
14	71/80	Cancer	Home hospital	Present	x	x	x	x	x		
15	81/90	Cancer	Nursing home	Last week	x		x	x	x	x	
16	51/60	Cancer	Hospital	2 months ago	x	x		x		x	
17	51/60	Amyotrophic lateral sclerosis	Home hospital	One year ago			x		x		
18	61/70	Cancer	Home hospital	Present	x	x		x	x		
19	61/70	Unknown	General practitioner	6 months ago	x	x	x		x	x	
20	51/60	Cancer	Hospital	1 month ago	x	x	x			x	x
21	61/70	Cancer	Hospital	6 months ago	x	x	x		x		
22	81/90	Cancer	General practitioner	15 days ago	x	x	x	x	x	x	
23	91/100	Old age	General practitioner	Present		x	x		x	x	x
24	51/60	Cancer	Hospital	15 days			x	x		x	
25	N/A	N/A	N/A	N/A	N/A	N/A	N/A	N/A	N/A	N/A	N/A
	(no situatiosn evocated)										

### Data analysis

Thematic analysis coupled with lexical analysis analysis identified three main role domains identified by the participants (see Table 3.). A first category was the drug expertise and clinical work implemented by professionals. A second area referred to the technical and administrative management of care. The third area was the psychosocial support provided by the participants to their patients at the end of life and to their families.

**Table 3:** Key elements of representations of participants’ role in palliative home care

Roles	Keywords representative of the domain (X^2^)**	Role elements
Drug expertise and clinical work	pain (252) food* (245) supplement (262) morphine (185) morphin* (111)	Dispensing of medicines, comfort care, nutrition, medical equipment
		Analysis of interactions, potential side effects
		Therapeutic education, monitoring of adherence
		Monitoring of symptom progression and adaptation to the clinical situation
Care management	HAD (home hospitalization) (175) order (111) doctor* (102) call (81) hospital (78) city (67)	Interactions with health professionals and structures, e.g., doctors, nurses, hospital, service provider
		Coordination of care
		Administrative and financial management
Psychosocial support for patients and/or caregivers	child* (103) died* (50) girl (45) end (39) mom (34) husband (33) spouse (26) dying (25)	Psychological support: presence, listening, comfort Social support: offer of services (e.g., home delivery, installation of equipment), sharing of resources (e.g., professionals, associations)

*Note*: * An asterisk at the end of a word indicates that this word was found in various forms in French—for example, in the feminine, masculine, plural, or singular forms; **The degree of co-presence of words in the verbatims is calculated by Alceste software using the normalized chi-square (X^2^), which determines the link that unites a word to a class

#### Drug expertise and clinical work

The theme of drug expertise and clinical work often represented the first theme addressed by participants. It develops the drug expertise and clinical work implemented by community pharmacists in a palliative or end-of-life home care setting. The words “dietary supplements” (“food*”; “supplement”), “morphine”, “morphin*” or “pain” made up a very large part of the lexicon in this category (see Table 3).

Support for patients in their feeding was a recurrent theme in the participants’ discourse. This support was mentioned by 18 of the 24 participants who spoke about their last end-of-life care (see Table 2). It was most frequently cited by participants as a trigger for their awareness that their patient required palliative care, firstly because they had learnt that the consequences of undernutrition are particularly harmful for cancer patients (see participant 8), and perhaps also because this factor is highly perceptible and time-consuming for a pharmacist (see participants 14 and 4):

“*Because if someone has been diagnosed with cancer, the only thing they need to keep their eyes on are the weight scales, and as soon as there’s the slightest sign of weight loss, you need to be on the alert straight away. The same goes for people with bedsores, who are bedridden and undernourished. Because we know that catabolism is more important in the metabolism.*” (Participant 8).

Nutritional support ranged from the delivery of hygienic and dietary advice to the installation of parenteral nutrition, as well as assistance in the choice of food supplements. Oral Nutritional Supplements (ONS) prescribed by doctors and delivered by community pharmacists give rise to many discussions with caregivers and/or patients at the pharmacy counter. On the borderline with food and medicine, food products for special medical purposes take time and a strong commitment to deliver, as community pharmacists want their patients to test tastes, textures and colors before choosing the good supplements:

*“We have a whole range of products here [in the pharmacy], she [the patient] is still at the drinks, milk drinks or yoghurts stage, so it’s really a question of ‘What do you like?’ ‘What don’t you like?’ ‘Do you want fruit?’ ‘Do you like it sweet?’ ‘Salty?’ It’s up to her again, we have a whole range of products, she’s tested them and there are products she likes more than others; and then I think it’s like everyone else, when every day she eats something praline maybe afterwards she’s a bit fed up with praline, she wants to move on to… it’s summer perhaps with things that are a bit fruitier, a bit more in tune with the times.”* (Participant 14).

ONS are also heavy products that patients and/or caregivers can’t always take home on their own. They need help for transportation, and that’s sometimes what drives pharmacists to home delivery:

*“My colleagues had to make deliveries, because there were these famous nutrition bags, which were very heavy, and the lady didn’t really have a car, so it was a bit complicated for her, so my colleagues went several times, when we could, to deliver the bags to the lady’s home. "* (Participant 4).

Requests for pain relief were another trigger for participants’ awareness that a patient was potentially at the end of life and formed another recurring theme in 18 of the 24 participants’ verbatim reports (see Table 2). Most participants associated a patient’s palliative care needs with the presence of significant pain, which was more or less well managed by pharmacology. Only two participants indicated that pain management was not part of their role and that it was the sole responsibility of the prescribing physician. Most often, the community pharmacist tried to relieve the patient’s pain by proposing solutions to the patient, the caregiver, or the prescriber—for example, by suggesting changes in treatment or dosage. Four out of 24 participants took responsibility for dispensing analgesic treatments without a prescription, as described by participant 5:

*On a Saturday afternoon, there will be a patient who is hospitalized at home or whatever, who needs [fast-acting morphine sulfate] or whatever. There is no prescription. We know we will have the prescription, but theoretically we cannot give it, we cannot do anything. We will give him a few tablets to get him through the weekend*.

By law, community pharmacists must not dispense products that have not been prescribed by a doctor. But they are also the only professionals authorized to dispense painkillers and, as health professionals, they see their role as helping people in pain. In the end, when faced with a patient seeking relief without a prescription, or with a prescription that doesn’t comply with the law, the community pharmacist is often flexible, knowing that he or she will be able to rectify the situation within a few days, as he or she generally has good contacts with prescribers, as describe participants 15 and 24:

*“If the prescription is not compliant, I’m not going to say ‘come on, you’ll be back in 3 days and then the morphine will be available in 3 days’. No, it’s not like that. We’ve got a prescriber we know, who may not have prescribed correctly, but we’re not going to let the patient suffer for 3 days because the prescription is wrong. So, these are liberties we’re taking in terms of regulations, but humanly speaking, it’s out of the question for us to do otherwise. It means that, yes, we’ll put them on hold, we’ll dispense the products and then, in any case, we can wait 24–48 hours before we have this information, before we have the right prescription, properly filled. But the patient has his treatments, and we start the painkiller, and we start this, we start that. What I’m telling you is almost a daily occurrence.”* (Participant 15).

“*Anything involving morphine, where we don’t have the prescriptions in order and so, well, it’s managed on a daily basis, but we call the doctors and they rewrite the prescriptions for us. (…/…) It happens a lot.*” (Participant 24).

In addition to relieving pain, participants generally monitored the evolution of their patients’ condition and were attentive to the appearance of new symptoms and sometimes recommended possible changes to treatment to the patient himself, to the caregiver or directly to the prescriber. Therapeutic education of patients and/or their families was also mentioned in 15 of the 24 verbatims. After a consultation with their specialist (e.g., oncologist, neurologist), patients had rarely assimilated all the information concerning their treatment and came to the pharmacy counter with a strong need for explanations:

*“…and then we see how well she has understood, depending also on what was said at the CHU [University Hospital Centre], because it is a lot of information and not everyone is able to understand. So, we go over it together, we see what chemotherapy is going to be done and then the side effects*” (Participant 8).

Seven of the 24 participants (see Table 2) also monitored their patients’ compliance. This vigilance was implemented when the community pharmacist went to the patient’s home for a delivery, for example, or at the pharmacy counter:

“*We can see, in relation to the boxes, if they need to be renewed or not, we can already know if at least they are taking their treatment correctly or not; or the opposite, if they are taking too much or not enough. So already in relation to the delivery, we can see if it is balanced or not*” (Participant 23).

#### Care management

A second theme identified in the qualitative analysis was care management around the patient via interactions with other health professionals, hospital structures, equipment or service providers, or billing organizations. The important presence of this theme in the verbatims was also highlighted by Alceste software. In this thematic class, there was significant occurrence of the terms “hospitalization at home”, “prescription”, “physician*”, “call”, “hospital”, or “city”.

In general, participants saw palliative care as a multidisciplinary effort in which community pharmacists were involved to varying degrees. The verbatim reports revealed that the organization of palliative care at home varies greatly from one area to another (e.g., rural or urban context) or from one situation to another (e.g., a young cancer patient receiving hospital follow-up, or a multi-pathological elderly patient followed by his or her general practitioner). Of the 24 cases reported by the participants, medical follow-up was provided by a general practitioner in eight situations, by a hospital in eight situations and by a home hospitalization structure in seven situations, while one patient benefited from the medical follow-up of an institution for dependent elderly people (Etablissement d’hébergement pour personnes âgées dépendantes; see Table 2.). The prescriber was not always the care manager and the participants did not always identify a person or structure responsible for coordination (e.g., general practitioner, hospital structure, private nurse). In these different contexts, community pharmacists sometimes acted as relays between the different health professionals, as described by this participant:

“*There is a lady at home. The nurse comes to see us and says, ‘I think she might need … well, it would help us if we had a patient lift, a sit-to-stand device, or whatever’, and we say, ‘ok, we’ll see with the doctor if he can give us a prescription and then we’ll have it delivered, we’ll call the provider’*” (Participant 20).

Located at the interface of their patients’ homes, doctors’ and nurses’ offices, hospitals, and equipment providers, community pharmacists often informally assumed the role of care coordinator. Exchanges of information between the various care providers could take place at the pharmacy (often with nurses), by telephone or email (often with doctors), or by fax with hospitals.

#### Psychosocial support for patients and their families

The important presence of theme of psychosocial support for patients and their families in the verbatim shows the importance given by the participants to the psychosocial support they offer their patients and/or their entourage in the specific context of the end of life. In this category, we find high occurrence of words belonging to the lexical field of family—“child”, “daughter”, “mom”, “husband”, or “spouse”—as well as terms referring to death: “deceased”, “die”, “end”.

Nineteen out of 24 participants reported offering psychosocial support to their patients at the end of life (see Table 2). The pharmacies were described as community-based, open to all residents of a neighborhood, walk-in, with no appointment necessary. Participants speak of long-lasting relationships with their patients, based on the multiplication of services requested from the community pharmacist in response to everyday health problems, as this dialogue between the interviewer and the participant 15 shows:

*Interviewer: -He calls you by your first name? - Interviewee: Yes. - How well do you know him? - We’re not intimate, he knows me because he’s been coming here for 10 years.*

These long-term relationships form the basis of a bond that is regularly described as close:

*“For Christmas, this lady always left us an orchid or, yes, she did, when you stay in the same place for over 20 years, that’s for sure they give us something. Especially for the elderly. We’re always the same age as their grandchildren, so they establish a kind of closeness a little bit. We replace those who aren’t there.”* (participant 9).

This long-term relationship is often identified as the factor that triggers the pharmacist’s offer of emotional support to the patient, as illustrated by this participant:

*“There are quite a few where we’ve been following them for a very long time. So, there are things too, I don’t know, they can confide in or say things that they wouldn’t say to someone, not a stranger but someone they don’t know”* (Participant 2).

Another major role described by the participants was that of welcoming and listening to the caregivers. Support for family members was mentioned by 15 participants (see Table 2). Caregivers were often described as isolated, suffering, and in need of an attentive and understanding professional ear, as describe participant 4 and 12 :

“*But in this support, it wasn’t him [the patient] I felt I was following, it was rather her [the caregiver] who came, pfff… not every day, but almost, to the pharmacy to get her medication.”* (participant 4).

*“Interviewee: His wife was in charge of getting the drugs here, and it was a nice way for her to get out and see us. - Interviewer: You think she liked coming here? - Interviewee: Yes, yes, absolutely. - Interviewer: Why do you think she liked it? - Interviewee: Because there’s contact, it gets her out, when you’re treating someone it’s very difficult, so it’s good to get out and see other people.”* (participant 12).

The support offered to them by the participants consisted mainly of sympathetic listening to their daily difficulties. These informal exchanges, which varied greatly in length depending on the needs of the caregivers and/or the context of the pharmacy, often took place at the pharmacy counter and sometimes in the patient’s home:

*“[We would deliver to the home] systematically, I would say almost once a week, to maintain the link, to just take five minutes to talk with her husband [the caregiver] to see if at that time he needed to talk”* (Participant 1).

In addition to providing emotional support to their patients and/or their caregivers, community pharmacists also provided a form of social support in various ways, such as offering home delivery of treatments for patients who had difficulty moving or carrying heavy treatments (22 participants; see Table 2). They could also help install medical equipment in the patient’s home. Finally, patients and/or their families sometimes came to community pharmacists to seek other services or resources, such as contact information for a night sitter, a paramedic professional, or an association.

## Discussion

The aim of this study, which took the form of interviews with community pharmacists, was to examine community pharmacists’ perceptions of their role in home palliative care in France. The interviews indicated a variety of roles which could be divided into three domains: drug expertise and clinical analysis, care management, and psychosocial support for the patient and his/her family. These results confirm the participation of community pharmacists in the objectives set by the WHO: “Palliative care improves the quality of life of patients and that of their families who are facing challenges associated with life-threatening illness, whether physical, psychological, social or spiritual.” [1]. By positioning themselves at the heart of the distinct systems of medication and psychosocial support for patients and their families, community pharmacists are indeed seen as extended actors in palliative care. While the elements that concern community pharmacists’ medication expertise and care management align with the missions of pharmacists in palliative care described in the literature [2, 5, 15, 16] and recommendations issued by the ASHP [3], psychosocial support for patients and their caregivers, described by participants as a central focus of their role, has remained little considered until now. This element of psychosocial support may be facilitated by the relational continuity that appears to exist between community pharmacists and their patients. Relational continuity of care (COC) is defined as “a long-term, ongoing therapeutic relationship that connects different healthcare episodes” [17]. In terms of physician-patient COC, this special relationship leads to improvements in prevention, chronic disease management, and patient/physician satisfaction [18]. Community pharmacist-patient COC, on the other hand, has been largely unstudied [17]. Choi and Lee’s (2022) [17] meta-analysis on the subject concludes that the only studies on community pharmacist-patient COC, conducted in Canada and the United States, show a high degree of relational continuity between community pharmacists and patients attending only one pharmacy. Our study, which took place in France, in turn indicates a long-term relationship between community pharmacists and their patients felt by our participants.

The psychosocial support offered by community pharmacists could be particularly helpful for patients’ loved ones. These caregivers are at higher risk than the general population of developing anxiety-depressive disorders [19, 20]. Our study indicates that community pharmacists consider themselves to be on the front line of these suffering individuals. Meetings between community pharmacists and caregivers are indeed frequent. They occur when the latter come to collect medication for their loved ones who can no longer travel. Our participants identify these visits to the pharmacy counter as being among the rare moments when caregivers are face to face with a health professional in the absence of the patient. This contextual window of opportunity is used by community pharmacists to support caregivers and is a valuable feature that could probably be put to even better use if this support were more clearly identified and encouraged.

Our verbatim reports also indicate that community pharmacists provide therapeutic education to their patients, particularly cancer patients—for example, when they educate them about their chemotherapy treatments. Professionals have a variety of tools at their disposal, easily accessible online, that give them access to materials and practices related to the quality, safety, and efficiency of therapies (e.g., patient follow-up sheets [21]). These tools allow them to both train and inform their patients. Community pharmacists also allow themselves flexibility in dispensing medications when it comes to relieving patients’ pain even when they do not have a compliant prescription. This flexibility in dispensing may be an echo among professionals of the French law on the rights of patients and users of the health system, which stipulates that “everyone has the right to a dignified end of life accompanied by the best possible relief of suffering. Health professionals shall use all means at their disposal to ensure that this right is respected” [22]. Whatever the reasons for the professionals’ decisions, this flexibility seems to be part of the consequences of the relationship of trust that is established over the long term between the community pharmacist and the prescribers and between the community pharmacist and their patients. This trusting relationship between the community pharmacist and the patient and/or family member as described by the participants could be a lever to improve patient compliance. Wilson et al. (2020) [23] looked at medication management by palliative home care patients. Their qualitative study was based on the experiences of healthcare professionals in Great Britain, including those of community pharmacists. The authors found that few healthcare professionals provide real support to patients in adhering to their medication, and that this responsibility actually falls to the patients themselves and their families and is often too heavy a burden for them. Wilson et al. (2020) [23] concluded that palliative care patients should have better follow-up in this area. In their meta-analysis of community pharmacist-patient COC, Choi and Lee (2022) [17] found that the presence of relational continuity between patients and community pharmacists could improve medication adherence and reduce inappropriate use of therapies. The potential existence of a relational continuity of care between French community pharmacists and their patients could be used as a resource to enable the patients in palliative care to better follow their treatments. From this point of view, the recent introduction in France of “oral chemotherapy” interviews between patients taking oral anti-cancer drugs and their community pharmacist [24] also offers the prospect of better support for patients in palliative care. These scheduled interviews are designed to improve pharmaceutical support for patients undergoing oral anti-cancer treatment, by reinforcing the pharmacist’s role in advising and supporting patients in complying with their treatment, and in combating drug-related iatrogenicity.

A question about this study concerns end-of-life or palliative care situations reported by participants. In 17 out of 24 cases, cancer was the cause identified by the participants of their patient’s entry into the end of life. However, cancer deaths represent only 20.4% of all deaths at home in France [25]. This significant discrepancy between the number of cancers cited and the number of expected cancers may indicate that these situations require the services of community pharmacists more than others (e.g. cardiovascular diseases, age-related end-of-life). Another hypothesis is that cancer is more strongly associated with death than other pathologies in participants’ representations. Whatever the reasons, it is worth emphasizing the strong presence of cancer in the verbatim reports, compared to other causes of death at home. This study questioned community pharmacists about their own perception of their role. It will certainly be necessary to question other stakeholders, notably patients, caregivers and other health professionals, in order to obtain a broader vision of the roles of community pharmacists in home end-of-life care.

Another limitation of our study is the nature of the study population. A total of 66 community pharmacists were contacted to obtain 27 appointments, an acceptance rate of 41%. The interviewer’s request was costly for participants in terms of time, since the interviews were scheduled to last 45 min. This suggests that the participants who agreed to receive the interviewer in their pharmacy were genuinely interested in the subject of this study. The representativeness of their responses needs to be explored in a quantitative larger study.

## Conclusion

This study highlights the wide variety of roles adopted by French community pharmacists in home palliative care. Some of these roles, such as psychosocial support for patients and their caregivers, have been little described until now and are unrecognized. Given the need of health professionals trained in palliative care at home, community pharmacists could be better identified and integrated into these systems. Further studies involving other stakeholders, including patients, carers and other healthcare professionals, are needed to gain a broader view of the role of dispensing pharmacists in end-of-life care at home.

### Electronic supplementary material

Below is the link to the electronic supplementary material.

Supplementary Material 1

Supplementary Material 2

Supplementary Material 3

Supplementary Material 4

Supplementary Material 5

## Data Availability

Data generated and/or analyzed during the current study are stored in a database that meets the quality criteria of CIC 1431. Only the investigator who led the conduct of the study can store the link between the anonymization code and the full identity of the participants. The rest of the data is available from the corresponding author upon reasonable request.

## Abbreviations

WHO: World Health Organization
ASHP: The American Society of Health-System Pharmacists
COC: Continuity of Care
ONS: Oral Nutritional Supplements
