# Supplementary material for: The roles of French community pharmacists in palliative home care
Source: BMC Palliat Care. 2024 Mar 23;23:79. doi: 10.1186/s12904-024-01406-6 (PMC10960433; doi:10.1186/s12904-024-01406-6)
Supplement: Supplementary file 1 — Supplementary Material 1 [file 12904_2024_1406_MOESM1_ESM.pdf]

UNIVERSITÉ
Clermont
Auvergne

Clermont-Ferrand, le 04 octobre 2021

Mme Axelle VAN LANDER
Unité de Recherche AutomédiCation
aCcompagnement Pluriprofessionnel
PatienT (ACCePPT)
Université Clermont Auvergne

Nos Réf. : IRB00011540-2021-60

Madame,

Vous nous avez sollicités à propos d'un projet intitulé :

« Analyse qualitative de la perception qu'ont les pharmaciens de leur rôle dans l'accompagnement des patients à domicile en soins palliatifs »

Vous nous avez précisé que cette étude a pour objectif d'explorer, à travers une série d'entretiens, le fait que les pharmaciens d'officine ont un rôle essentiel d'accompagnement des patients et de leur entourage.

Cette étude ne soulève pas de problème éthique particulier et ne relève pas du domaine d'application de la réglementation régissant les recherches impliquant la personne humaine, au sens de l'Article L.1121-1-1 et l'Article R.1121-1 du code de la santé publique.

Nous attirons néanmoins votre attention sur le fait que, dans ce contexte, du fait de l'enregistrement des différentes données et informations, il vous appartient de vous renseigner auprès du Délégué à la Protection des Données de l'UCA (dpd@uca.fr) sur les obligations liées aux déclarations auprès de la CNIL.

Veuillez agréer, Madame, l'expression de nos salutations distinguées.

Sophie Monceau
Pour la Directrice du Comité d'Éthique de
la Recherche IRB-UCA
Anne FOGLI

Comité d'Ethique de la Recherche IRB-UCA
49 Boulevard François Mitterrand - CS 60032 – 63 001 Clermont-Ferrand
Contact : irb@uca.fr

UNIVERSITÉ
Clermont
Auvergne

Clermont-Ferrand on October 04, 2021

Mrs Axelle VAN LANDER
Unité de Recherche AutomédiCation
aCcompagnement Pluriprofessionnel
PatienT (ACCePPT)
Université Clermont Auvergne

Nos Réf. : IRB00011540-2021-60

Madame,

You requested us about a project entitled :

« Analyse qualitative de la perception qu'ont les pharmaciens de leur rôle dans l'accompagnement des patients à domicile en soins palliatifs »

The French law on biomedical researches (Article L.1121-1-1 and Article R.1121-1 of the public health code) does not apply to this study, however, this one does not present particular ethical problem.

We call nevertheless your attention on the fact that, in this context, because of the recording of various data and informations, it is up to you to inquire about the obligations connected to the statements with the CNIL (NATIONAL COMMISSION FOR INFORMATION TECHNOLOGY AND CIVIL LIBERTIES)), by contacting the Data Protection Officer of the University (dpd@uca.fr).

Best Regards.

Sophie Monceau
Pour la Directrice du Comité d'Éthique de
la Recherche IRB-UCA
Anne FOGLI

Comité d'Ethique de la Recherche IRB-UCA
49 Boulevard François Mitterrand - CS 60032 – 63 001 Clermont-Ferrand
Contact : irb@uca.fr
