# Supplementary material for: The roles of French community pharmacists in palliative home care
Source: BMC Palliat Care. 2024 Mar 23;23:79. doi: 10.1186/s12904-024-01406-6 (PMC10960433; doi:10.1186/s12904-024-01406-6)
Supplement: Supplementary file 2 — Supplementary Material 2 [file 12904_2024_1406_MOESM2_ESM.pdf]

APPENDIX 2 - Information and non-objection form given to participants

ACCePPT
AutomédiCation aCcompagnement Pluriprofessionnel PatienT

Director, Professor Brigitte VENNAT,

UFR Pharmacy - University of Clermont Auvergne

Deputy Director - Professor Philippe VORILHON

UFR Medicine - University of Clermont Auvergne

Referents :

Chantal SAVANOVITCH, NUANCe referent

Hélène VAILLANT-ROUSSEL, DECIdE referent

Axelle VAN LANDER, ACCOMPALLIA referent

INFORMATION AND NON-OBJECTION FORM

Principal investigator	ACCePPT UCA Laboratory<br>Axelle Van Lander<br>UFR of Pharmacy<br>TSA 50400<br>28, Place Henri Dunant<br>63001 CLERMONT-FERRAND<br>Email : avanlander@chu-clermontferrand.fr
Promoter	DRCI CHU Besançon<br>Professor Régis Aubry
Co-investigator	Isabelle Cuchet, PhD student in psychology

The ACCePPT laboratory of the University of Clermont Auvergne, in collaboration with the University Hospital of Besançon, is currently conducting a research in Human and Social Sciences, entitled: PHARMAPAL, the role of community pharmacists in supporting patients in palliative care at home.

This exploratory study aims to determine the role of the community pharmacists in in the follow-up of palliative care patients at home. It has received financial support from the Fondation de France and will last for three years.

Community pharmacists and assistant pharmacists in rural and urban areas in the Auvergne-Rhône-Alpes region, will be met individually at their place of work, ideally in a confidential area of their pharmacy. The interviews, lasting on average 45 minutes, will be recorded. Their contents will be transcribed in full and anonymized for analysis. The data collected may be the subject of a report and/or a scientific publication which will not mention the identity of the participants.

You are a pharmacist in the Auvergne-Rhône-Alpes region. As such, you regularly accompany patients with serious, progressive or terminal illnesses and their families. We hereby ask you not to object to your participation to an individual interview which will aim to collect your perceptions of this role.

You are free to accept or refuse to participate in this interview. You will not be paid for your participation. The constraints of your participation consist in meeting the study co-investigator at your

pharmacy for 45 minutes. The expected benefits of this study are the advancement of research and a better recognition of the recognition of the community pharmacist's role in palliative care.

You can ask for further explanations at any time or withdraw your participation agreement at any time. For any questions after the interview, you can contact the principal investigator, Axelle Van Lander at 04 73 750 960 or by email : avanlander@chuclermontferrand.fr

Once you have read this note and obtained the answers to your questions, you will be given the opportunity to formulate your opinion, you will be asked to formulate your non-opposition by signing this document.

Date: .../.../...

Signature of the participant, preceded by the words "read and understood

....

Signature of the co-investigator :

....

ACCePPT

AutomédiCation aCcompagnement Pluriprofessionnel PatienT

ANNEXE 2 – Formulaire d'information et de non opposition donné aux participants

Directeur, Professeur Brigitte VENNAT,

UFR de Pharmacie - Université Clermont Auvergne

Directeur adjoint - Professeur Philippe VORILHON

UFR de Médecine – Université Clermont Auvergne

Référents :

Chantal SAVANOVITCH, referent NUANCe

Hélène VAILLANT-ROUSSEL, référent DECIdE

Axelle VAN LANDER, référent ACCOMPALLIA

FORMULAIRE D'INFORMATION ET DE NON OPPOSITION

Investigateur principal	Laboratoire ACCEPPT UCA<br>Axelle Van Lander<br>UFR de Pharmacie<br>TSA 50400<br>28, Place Henri Dunant<br>63001 CLERMONT-FERRAND<br>Email : avanlander@chu-clermontferrand.fr
Promoteur	DRCI CHU Besançon<br>Professeur Régis Aubry
Co-investigateur	Isabelle Cuchet, doctorant en psychologie

Le laboratoire ACCePPT de l'université Clermont Auvergne, en lien avec le CHU de Besançon, mène actuellement une recherche en Sciences Humaines et sociales, intitulée : *PHARMAPAL, le rôle des pharmaciens dans l'accompagnement des patients à domicile en soins palliatifs.*

Cette étude exploratoire a pour objectif de déterminer la place du pharmacien d'officine dans le suivi des patients en soins palliatifs à domicile. Elle a reçu le soutien financier de la Fondation de France et s'étalera sur trois ans.

Des pharmaciens, titulaires et adjoints d'officine, répartis en zone rurale et urbaine dans la région Auvergne-Rhône-Alpes, seront rencontrés individuellement sur leur lieu de travail, idéalement dans une zone confidentielle de leur officine. Les entretiens, d'une durée moyenne 45 minutes, seront enregistrés. Leurs contenus seront retranscrits intégralement et anonymisés pour être analysés. Les données recueillies pourront faire l'objet d'un rapport et/ou d'une publication scientifique qui ne feront pas état de l'identité des participants.

Vous êtes pharmacien d'officine en région Auvergne-Rhône-Alpes. A ce titre, vous accompagnez régulièrement des patients atteints de maladie grave, évolutive ou terminale et leur famille. Par la

présente, nous vous demandons votre non-opposition à votre participation à un entretien individuel qui aura pour but de récolter vos perceptions de ce rôle.

Vous êtes libre d'accepter ou de refuser de participer à cet entretien. Vous ne serez pas rémunéré pour votre participation. Les contraintes de votre participation consistent à rencontrer à votre officine le co-investigateur de l'étude pour une durée de 45 minutes. Les bénéfices attendus de cette étude sont l'avancée de la recherche et une meilleure reconnaissance du rôle du pharmacien en soins palliatifs.

Vous pouvez demander à tout moment des explications complémentaires ou retirer votre accord de participation. Pour toutes questions à l'issue de l'entretien, vous pouvez contacter l'investigateur principal, Axelle Van Lander au 04 73 750 960 ou par email : avanlander@chu-clermontferrand.fr

Lorsque vous aurez lu cette note et obtenu les réponses aux questions que vous vous posez, il vous sera proposé de formuler votre non-opposition en signant ce document.

Date: .../.../...

Signature du participant, précédé de la mention « lu et compris »

....

Signature du co-investigateur

....
