# Supplementary material for: The roles of French community pharmacists in palliative home care
Source: BMC Palliat Care. 2024 Mar 23;23:79. doi: 10.1186/s12904-024-01406-6 (PMC10960433; doi:10.1186/s12904-024-01406-6)
Supplement: Supplementary file 3 — Supplementary Material 3 [file 12904_2024_1406_MOESM3_ESM.docx]

**Consolidated criteria for reporting qualitative studies (COREQ): 32-item checklist**

| **Topic** | **Item n°** | **Guide questions/description** | **Answer** |
| --- | --- | --- | --- |
| **Domain 1: Research team and reflexivity** | | | |
| Personal Characteristics | | | |
| Interviewer/facilitator | 1. | Which author/s conducted the interview or focus group? | Isabelle Cuchet |
| Credentials | 2. | What were the researcher’s credentials? E.g. PhD, MD | PhD student |
| Occupation the study? | 3. | What was their occupation at the time of the study? | Research psychologist |
| Gender | 4. | Was the researcher male or female? | Female |
| Experience and training | 5. | What experience or training did the researcher have? | training in qualitative and quantitative research/ training in conducting interviews |
| Relationship with participants | | | |
| Relationship established | 6. | 6. Relationship established Was a relationship established prior to study commencement? | No |
| Participant knowledge of the interviewer | 7. | What did the participants know about the researcher? e.g. personal goals, reasons for doing the research | participants were told the interviewer was a researcher in psychology |
| Interviewer characteristics | 8. | What characteristics were reported about the interviewer/facilitator? e.g. Bias, assumptions, reasons and interests in the research topic | The interviewer explained the reasons and interests in the research topic |
| **Domain 2: study design** | | | |
| Theoretical framework | | | |
| Methodological orientation and Theory | 9. | What methodological orientation was stated to underpin the study? e.g. grounded theory, discourse analysis, ethnography, phenomenology, content analysis | thematic analysis plus lexical analysis (inspired by phenomenological analysis) |
| Participant selection | | | |
| Sampling | 10. | How were participants selected? e.g. purposive, convenience, consecutive, snowball | purposive sample with maximum variation |
| Method of approach | 11. | How were participants approached? e.g. face-to-face, telephone, mail, email | Participants were contacted first by email then by telephone |
| Sample size | 12. | How many participants were in the study? | 66 pharmacies were contacted |
| Non-participation | 13. | How many people refused to participate or dropped out? Reasons? | 40 people refused to take part: no time for the interviewer (45 minutes face-to-face requested) |
| Setting | | | |
| Setting of data collection | 14. | Where was the data collected? e.g. home, clinic, workplace | Each participant’s pharmacy |
| Presence of non-participants | 15. | Was anyone else present besides the participants and researchers? | No, but the interviews could be interrupted by participants’ colleagues or by phone calls for the participants |
| Description of sample | 16. | What are the important characteristics of the sample? e.g. demographic data, date | All participants were community pharmacists, working in Auvergne Rhône Alpes area (France). Data collected between 21 April and 5 August 2021 |
| Data collection | | | |
| Interview guide | 17. | Were questions, prompts, guides provided by the authors? Was it pilot tested? | An interview grid was developed and tested with three participants (community pharmacists) in a pilot study prior to the launch of the study. |
| Repeat interviews | 18. | Were repeat interviews carried out? If yes, how many? | No repeated interviews. |
| Audio/visual recording | 19. | Did the research use audio or visual recording to collect the data? | yes |
| Field notes | 20. | Were field notes made during and/or after the interview or focus group? | yes |
| Duration | 21. | What was the duration of the interviews or focus group? | interviews lasted an average of 53'32'' |
| Data saturation | 22. | Was data saturation discussed? | Yes, within the interviewer and one associate professor |
| Transcripts returned | 23. | Were transcripts returned to participants for comment and/or correction? | No |
| Domain 3: analysis and findings | | | |
| Data analysis | | | |
| Number of data coders | 24. | How many data coders coded the data? | 1 |
| Description of the coding tree | 25. | Did authors provide a description of the coding tree? | Yes, within the interviewer and one associate professor |
| Derivation of themes | 26. | Were themes identified in advance or derived from the data? | derived from the data |
| Software | 27. | What software, if applicable, was used to manage the data? | Excel, N'Vivo and Alceste softwares |
| Participant checking | 28. | Did participants provide feedback on the findings? | No, apart from the three participants of the pilot study |
| Reporting | | | |
| Quotations presented | 29. | Were participant quotations presented to illustrate the themes / findings? Was each quotation identified? e.g. participant number | Yes. Quotations were identified with participant number |
| Data and findings consistent | 30. | Was there consistency between the data presented and the findings? | yes |
| Clarity of major themes | 31. | Were major themes clearly presented in the findings? | yes |
| Clarity of minor themes | 32. | Is there a description of diverse cases or discussion of minor themes? | yes |
