# Supplementary material for: The roles of French community pharmacists in palliative home care
Source: BMC Palliat Care. 2024 Mar 23;23:79. doi: 10.1186/s12904-024-01406-6 (PMC10960433; doi:10.1186/s12904-024-01406-6)
Supplement: Supplementary file 4 [file 12904_2024_1406_MOESM5_ESM.odt]

Supplementary file

Interview grid

Primer Questions:

Can you tell me about the last time you cared for a palliative care patient at home?

If you have not done so, can you tell me about the last time you cared for an end-of-life patient at home?

Where and how did your care take place?

What was your role as a pharmacist?

Targeted questions:

| Objective | Questions |
| --- | --- |
| Awareness of the patient situation Definition of palliative care management | How did you identify that you were in a palliative care situation? Do you feel that you have ever been in a palliative care setting without knowing it? What do you think a palliative care management means? |
| Work demands, decision-making flexibility expectations of colleagues, patients, family and friends Relations with the other professionals | Do you feel free to decide how you do your work? Who was/were your main interlocutors in this palliative care management? How would you rate the quality of your relations with the professionals you worked with? Did you feel supported by your colleagues in the pharmacy/the other actors of the care? |
| Relations with the patients | Did you know your patient before they entered palliative care ? Did your palliative care management change your relationship with your patient or his/her family? What happened to your relationship with your patient or his or her family during and after your care? |
| Covid-19 | What has COVID-19 changed in the way you work? |
| Job satisfaction | Can you tell me and explain to me the main difficulty and the main satisfaction of your work in palliative care? |

Additional Question:

Is the palliative management you just described representative of your previous palliative care management? If not, could you tell me about another palliative management that went less well/better/differently?
